# A multi-treatment experimental system to examine photosynthetic differentiation in the maize leaf

**DOI:** 10.1186/1471-2164-8-12

**Published:** 2007-01-09

**Authors:** Ruairidh JH Sawers, Peng Liu, Katya Anufrikova, JT Gene Hwang, Thomas P Brutnell

**Affiliations:** 1 Boyce Thompson Institute, Cornell University, Tower Road, Ithaca, NY 14853, USA; 2 Department of Biological Statistics and Computational Biology, Cornell University, Ithaca, NY 14853, USA; 3 Department of Mathematics and Department of Statistics, Cornell University, Ithaca, NY 14853, USA; 4 Department of Plant Molecular Biology, Biophore, University of Lausanne, CH-1015 Lausanne, Switzerland; 5 Department of Statistics, Iowa State University, Ames IA 50011, USA

## Abstract

**Background:**

The establishment of C_4 _photosynthesis in maize is associated with differential accumulation of gene transcripts and proteins between bundle sheath and mesophyll photosynthetic cell types. We have physically separated photosynthetic cell types in the leaf blade to characterize differences in gene expression by microarray analysis. Additional control treatments were used to account for transcriptional changes induced by cell preparation treatments. To analyse these data, we have developed a statistical model to compare gene expression values derived from multiple, partially confounded, treatment groups.

**Results:**

Differential gene expression in the leaves of wild-type maize seedlings was characterized using the latest release of a maize long-oligonucleotide microarray produced by the Maize Array Project consortium. The complete data set is available through the project web site. Data is also available at the NCBI GEO website, series record GSE3890. Data was analysed with and without consideration of cell preparation associated stress.

**Conclusion:**

Empirical comparison of the two analyses suggested that consideration of stress helped to reduce the false identification of stress responsive transcripts as cell-type enriched. Using our model including a stress term, we identified 8% of features as differentially expressed between bundle sheath and mesophyll cell types under control of false discovery rate of 5%. An estimate of the overall proportion of differentially accumulating transcripts (1-π_0_) suggested that as many as 18% of the genes may be differentially expressed between B and M. The analytical model presented here is generally applicable to gene expression data and demonstrates the use of statistical elimination of confounding effects such as stress in the context of microarray analysis. We discuss the implications of the high degree of differential transcript accumulation observed with regard to both the establishment and engineering of the C_4 _syndrome.

## Background

Photosynthesis in the majority of plants occurs in a single photosynthetic cell type (C_3 _photosynthesis) [1]. Within the chloroplasts, the enzyme ribulose-1, 5-bisphosphate carboxylase/oxygenase (Rubisco) fixes atmospheric carbon by addition of CO_2 _and water to the five-carbon sugar ribulose-1, 5-bisphosphate (RuBP). Rubisco will also catalyze the oxidation of RuBP in a process known as photorespiration that does not fix carbon [2]. The reduction in efficiency associated with photorespiration and the energetic costs of recycling its products has been estimated to limit the performance of C_3 _photosynthesis by as much as 30% in hot arid conditions [3]. A number of taxa utilize a two-step carbon fixation process, known as C_4 _photosynthesis, to limit the impact of photorespiration upon photosynthetic performance [4]. Plants that utilize C4 photosynthesis appear to be at a particular fitness advantage under conditions of limited water availability, high temperature and high irradiance light [5]. Interestingly, some of the most promising grasses for biofuel production are C4 grasses, including Miscanthus × giganteus (Giant Miscanthus), *Panicum virgatum *(switchgrass), *Zea mays *(maize), *Sorghum bicolor *(sorghum) and *Saccharum officinarum *(sugarcane).

**Figure 1:**
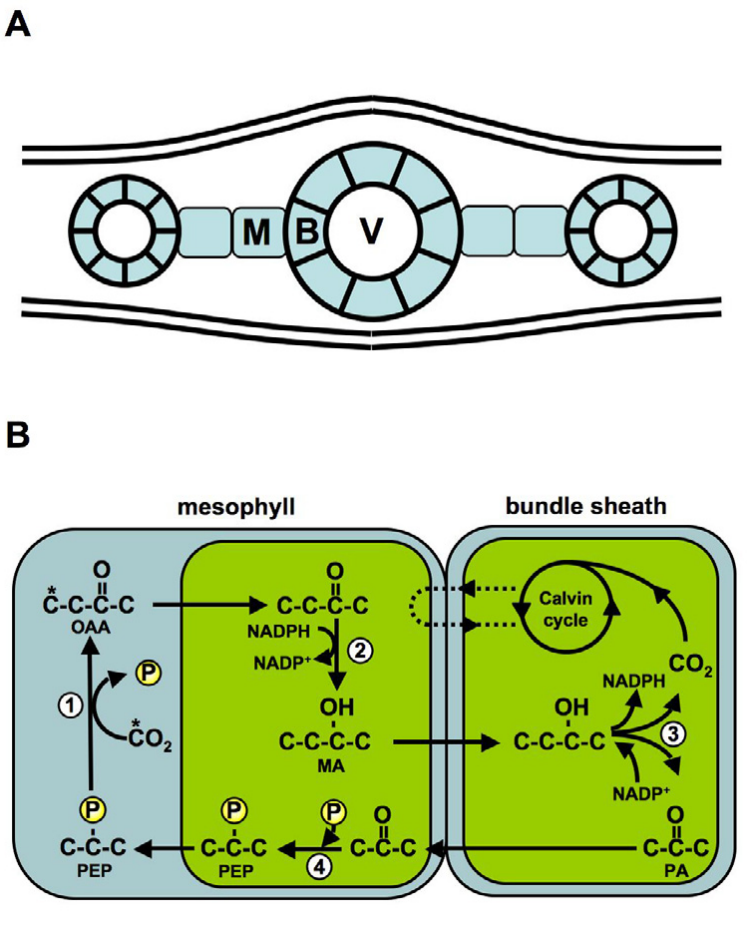
**C_4 _photosynthesis in the maize (*Zea mays*) leaf**. **A. **Schematic of a longitudinal cross section through a maize leaf showing Kranz anatomy. Thick-walled bundle sheath (B) cells surround longitudinal veins (V). Mesophyll cells (M) occupy the leaf space between vascular bundles. **B. **Major reactions of the C_4 _carbon shuttle. 1) Carbon is initially added to phosphoenolpyruvate (PEP) to form oxaloacetate (OAA) by the enzyme PEP carboxylase (PEPC). 2) OAA is transported to the M chloroplasts where it is reduced to malate (MA) by NADP-specific malate dehydrogenase (NADP-MDH). 3) MA is transported to the B where it is decarboxylated by NADP-malic enzyme (NADP-ME) to yield pyruvate (PA) and release CO_2 _to the Calvin cycle. 4) PA is returned to the M where it is phosphorylated by phosphoenol pyruvate dikinase (PPdK) to regenerate PEP and continue the cycle.

In C_4 _plants, Rubisco accumulation is spatially restricted to CO_2_-rich sites within the leaf so that the carboxylase reaction is favoured over photorespiration. In maize, Rubisco accumulation is restricted to thick-walled bundle sheath (B) cells that surround the leaf veins (Figure 1A). Carbon is initially fixed in adjacent mesophyll (M) cells and subsequently transported, by a multi-enzyme carbon shuttle, into the B, where decarboxylation elevates local CO_2 _levels and generates an environment for efficient Rubisco function (Figure 1B).

Cell-type specific differences in morphology and physiology are fundamental to C_4 _photosynthesis [1,6]. Detailed analysis of B and M differentiation in maize has shown that Rubisco, enzymes of a C_4 _carbon shuttle and components of the light-harvesting machinery accumulate to different levels in B and M cells [7]. B cell chloroplasts are predominately agranal and do not accumulate key components of the water oxidizing complex of photosystem II (PSII) [8,9]. Consequently, a number of processes requiring chemical reduction, including portions of Calvin cycle [10,11], synthesis of antioxidants [12] and nitrogen assimilation [13] are localized to the M cells. Despite detailed understanding of certain metabolic pathways utilized in C_4 _photosynthesis, the molecular mechanisms governing cell differentiation and the full extent of metabolic partitioning are still to be fully characterized. Promoter fusion, methylation assays and transient expression studies have identified a number of *cis *acting elements in the promoter sequences of C_4_-related genes [14-17]. Much less is known about *trans *acting factors that may drive the C_4_differentiation process [18,19]. Genetic approaches have resulted in the isolation of maize mutants characterized by B cell-specific defects, but these mutants have not directly identified regulators of cell-specific development [20-22].

Many biochemical and molecular studies of C_4 _photosynthetic cell types have made use of techniques for isolation of separated cells. Typically, B cells have been isolated as vascular strands by mechanical disruption and M cells isolated as protoplasts by enzymatic digestion [23,24]. Therefore, different isolation protocols complicate the identification of differences between the two cell types. This is especially true when comparing the accumulation of RNA transcripts because changes can occur rapidly in response to the stresses of protoplast preparation [25]. When small numbers of genes have been analyzed, additional treatments have been used to control for the effect of these stresses (e.g. [22]). In contrast, previous multi-gene profiling studies of C_4 _cell types have not accounted for such effects in the initial analysis [26-28]. Following preliminary two-sample microarray experiments, we were especially concerned with the problem of mistakenly identifying stress-induced transcripts as M enriched (Sawers *et al*., unpublished observations).

In order to control for a stress effect associated with M cell isolation, total leaf and stressed total leaf samples were included in analysis. In general, microarray experiments are based on paired comparisons [29]. Multiple paired comparisons may be linked, as in a time course, or may be cross-referenced, as in a clustering analysis [29]. In the case of the isolated C_4 _cell types, the situation is somewhat different in that cell type and effects of the separation protocol are partially confounded between treatments. To formally describe this relationship, we have developed an analytical model to describe C_4 _gene expression and to allow elimination of the stress effect resulting from protoplast isolation. Using this approach, we have identified 1,280 features in the Maize Array Project oligonucleotide array that are predicted to be B or M enriched. We also present an analysis of the same data without consideration of the stress effect. Comparison of these two analyses demonstrates the importance of considering the stress effect and the application of a statistical modelling approach to control confounding factors in microarray experiments.

## Results and discussion

### Experimental design and data collection

**Figure 2:**
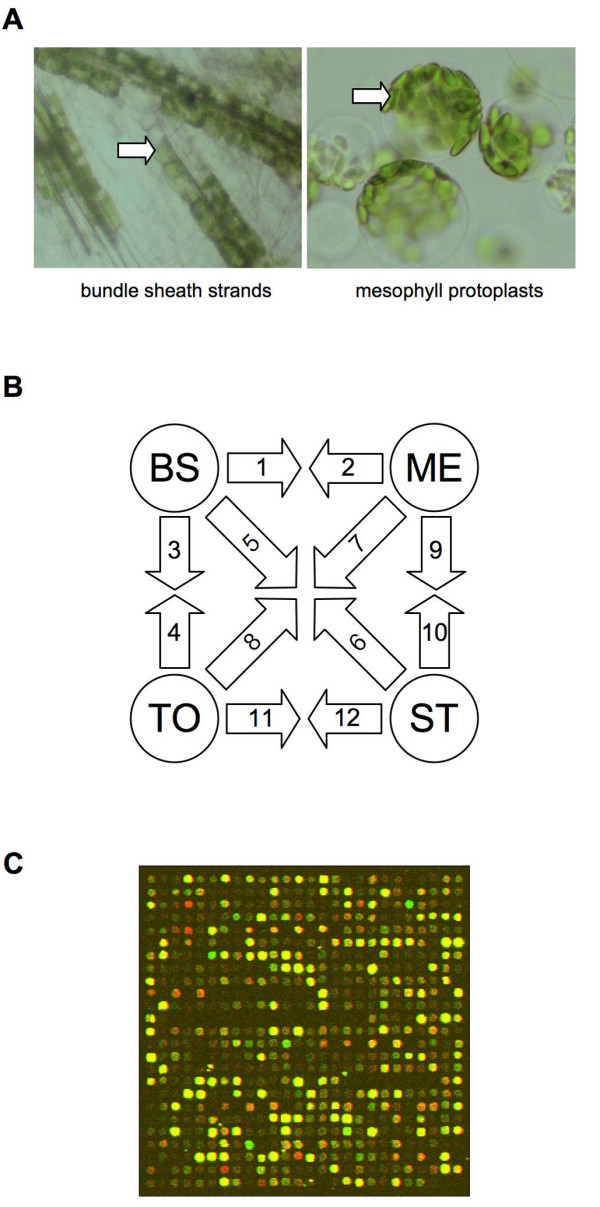
**Experimental design**. **A. **B stands and M protoplasts were isolated by mechanical disruption and enzymatic digestion, respectively. Representative chloroplasts are marked with an arrow. **B. **Interwoven loop design of microarray labelling. Four treatments (B, M, S, T) were compared using two-dye microarrays. Each arrow represents hybridization. RNA isolated from the treatment at the tail of the arrow was used to synthesize Cy3-labelled cDNA, and RNA isolated from the treatment at the head of the arrow was used to synthesize Cy5-labelled cDNA. Independent biological replicates were used for each labelling, giving a total of six per treatment. Numbers on arrows refer to Additional file 1. **C. **Pseudo-colour overlay of Cy3 and Cy5 images from a representative hybridization. A single 26 × 26 features sub-grid is shown. The complete University of Arizona oligonucleotide array consists of 96 such grids arrayed over two slides.

B strands (T_B_) and M protoplasts (T_M_) were isolated from 10 day-old W22 inbred maize seedlings by mechanical disruption and enzymatic digestion, as previously described [30] (Figure 2A). Isolation of M protoplasts required a 3 h enzymatic incubation that was not performed during isolation of B strands. To control for transcriptional differences arising from these different treatments, additional total leaf (T_T_) and total leaf stress (T_S_) samples were isolated. Both T_T _and T_S _samples contain a combination of B and M cells. Leaves for the T_T _sample were harvested and cut into thin strips as for the T_B _sample and then frozen directly in liquid nitrogen rather than subjected to mechanical disruption. Leaves for the T_S _sample were harvested and cut as for the T_M _sample, subjected to a 3 h mock enzymatic digestion and then frozen. An interwoven loop design [31] was used to directly compare all combinations of the four treatment groups (T_B_, T_M_, T_T_, T_S_) on two-label microarrays (Figure 2B). To maximize biological replication, a unique set of seedlings was used for each T_B_, T_M_, T_T _and T_S _replicate. The resulting design used 24 independent RNA samples analysed over 12 array sets.

Samples were analysed by hybridisation to a long-oligonucleotide microarray produced by the Maize Array Project consortium [32]. Maize sequences for design of this chip were predominantly selected from the The Institute for Genomic Research (TIGR) maize gene index [33]. To manufacture this microarray, approximately 50,000 sequences were positively orientated and identified as suitable for oligonucleotide design. This initial sequence set was supplemented with further EST sequences, organellar sequences, repeat sequences and community requests to provide a final design data set of 57,441 features. Single 70-mer oligonucleotides were designed for each sequence in the design set and printed over two glass slides.

Array detection (Figure 2C) and image analysis were performed as described in Methods. Images and intensity data may be accessed in a MIAME compliant form at the Maize Oligonucleotide Array Project website [34]. Data are also available in the NCBI GEO database, series record GSE3890. The data are presented in full on both the Maize Array Project and NCBI websites.

Feature intensity values were log-transformed and corrected for local background signal and a LOWESS procedure [35] was used to normalize between channels for each slide (see Methods). On the basis of the T_T _treatment (which contains both B and M), features with either low or saturating signal intensity were discarded (see Methods). A stringent filtering of low expression values was used to reduce the dimensionality of the data set in light of the complexity of the experimental design. High expression filtering was less stringent to avoid elimination of previously characterized, high abundance, C_4 _'marker' transcripts. Following filtering, 15,988 unique features were considered for subsequent analysis (Additional file 1). We considered this reduced data set appropriate for model development while maintaining sufficient and meaningful biological information to allow general conclusions on the extent of differentiation between B and M cell types.

### Construction of a model to include a stress term in the analysis of the B and M data set

The experimental design required the analysis of four, partially confounded treatment groups; *i.e. *the T_T _treatment contains both B and M cell types found in T_B _and T_M _treatments, while the T_M _treatment combines the effects of cell-specificity and the stress effect that is also seen in T_S_. In the following discussion, the term 'stress' is used to refer specifically to the effect of protoplast isolation on transcript levels. Importantly, although the aim of the experiment is to compare expression levels between B and M cell types, the level of gene expression within the intact M could never be directly measured because of the stress effect of M cell isolation. A model was constructed in order to formally describe this situation and to allow statistical elimination of the stress effect.

Let *V*_*1f *_and *V*_*2f *_represent the log_2 _transformed expression level of a given feature (*f*) in B and M cell types, respectively. The aim is to estimate (*V*_*1f *_- *V*_*2f*_) on a feature-by-feature basis. We use *c *to label the leaf sample, whether B cell prep (*c *= 0), M cell prep (*c *= 1) or total leaf sample (*c *= 2). The parameter *j *indicates the presence (*j *= 1) or absence (*j *= 0) of stress. For any given feature *f*, we fit the normalized signal *Y*_*fcjr *_with the model,

*Y*_*fcjr *_= (1 - *a*_*c*_)*V*_*1f *_+ *a*_*c*_*V*_*2f *_+ *jS*_*f *_+ ε_*fcjr*_   (1)

where *a*_*c *_is the proportion of M cells in the sample, *S*_*f *_represents the effect of stress on gene expression, *r *represents the replicate number from 1 through 6 and *f *represents the feature number from 1 to F, the number of features in the array. In this model, the effect of preparative stress is assumed to act additively and uniformly in both cell types. Below, the performance of the model will be assessed with respect to analysis in which the stress effect is not considered.

If we assume there is no contamination of the other cell type for the single cell treatments (T_B _or T_M_), the values of a_0 _and a_1 _(the proportion of M in the sample) are set to be 0 and 1, respectively. In practice, two types of cellular contamination might be recognized. First, a proportion of contaminating M cells will be present in the B prep and of B cells in the M prep. The level of this cross-contamination was estimated at below 5% as determined by semi-quantitative PCR using known markers for B (*RbcS *and *ChlMe*) and M cell identity (*Pepc *and *Mdh1*) (data not shown). The level of cross-contamination was considered to be sufficiently low as to be ignored, thereby simplifying the model by elimination of *V*_*1f *_and *V*_*2f *_terms in expressions describing M and B, respectively. The values of a_0 _and a_1 _could be adjusted to allow consideration of such contamination if desired. The presence of additional leaf cell types constitutes a second source of cellular contamination, perhaps most notably the inclusion of epidermal and vascular cells. For simplicity and economy, we do not consider this second source of contamination in our model. We estimated the value of a_2 _(the proportion of M cells in T_T _and T_S _preparations), by examination of leaf sections and by marker gene expression, at between 0.7 and 0.5 (data not shown). For the analysis presented here, the value of a_2 _was set at 0.5. The model (1) can thus be simplified as,

μ_*TBf *_= *V*_*1f*_  

μ_*TMf *_= *V*_*2f *_+ *S*_*f*_

μ_*TTf *_= (*V*_*1f *_+ *V*_*2f*_)/2

μ_*TSf *_= (*V*_*1f *_+ *V*_*2f*_)/2 + *S*_*f*_

where μ_*TBf*_, μ_*TMf*_, μ_*TTf *_and μ_*TSf *_represent expected expression levels indexed as appropriate to the four treatments T_B_, T_M_, T_T _and T_S _for feature *f*. Since not all treatments are observed within the same array, the spot-specific array effect is corrected as a random effect to the model (1) and estimated by REML [36]. The normalized values (see Methods) for each feature, corresponding to the 12 hybridizations, are listed in Additional file 1.

Two versions of the analysis were performed. First, an analysis as described above was used to generate estimates of (*V*_*1f *_- *V*_*2f*_) and *S*_*f *_for each feature (referred to as the stress model). Second, we repeated the analysis with the stress effect ignored (referred to as the simple model). A second set of estimates of (*V*_*1f *_- *V*_*2f*_) were calculated and compared with those obtained from the stress model. For both models, the deviation of each (*V*_*1f *_- *V*_*2f*_) estimate from 0 was investigated using a t-statistic. The q-value procedure of Storey et al. [37] was used to control the false discovery rate (FDR). The estimates of (*V*_*1f *_- *V*_*2f*_) and *S*_*f *_for the stress model are listed in Additional file 2 together with their test statistics. The estimates of (*V*_*1f *_- *V*_*2f*_) obtained from the simple model are listed in Additional file 3.

### Comparison of analyses using the stress and simple models

**Figure 3:**
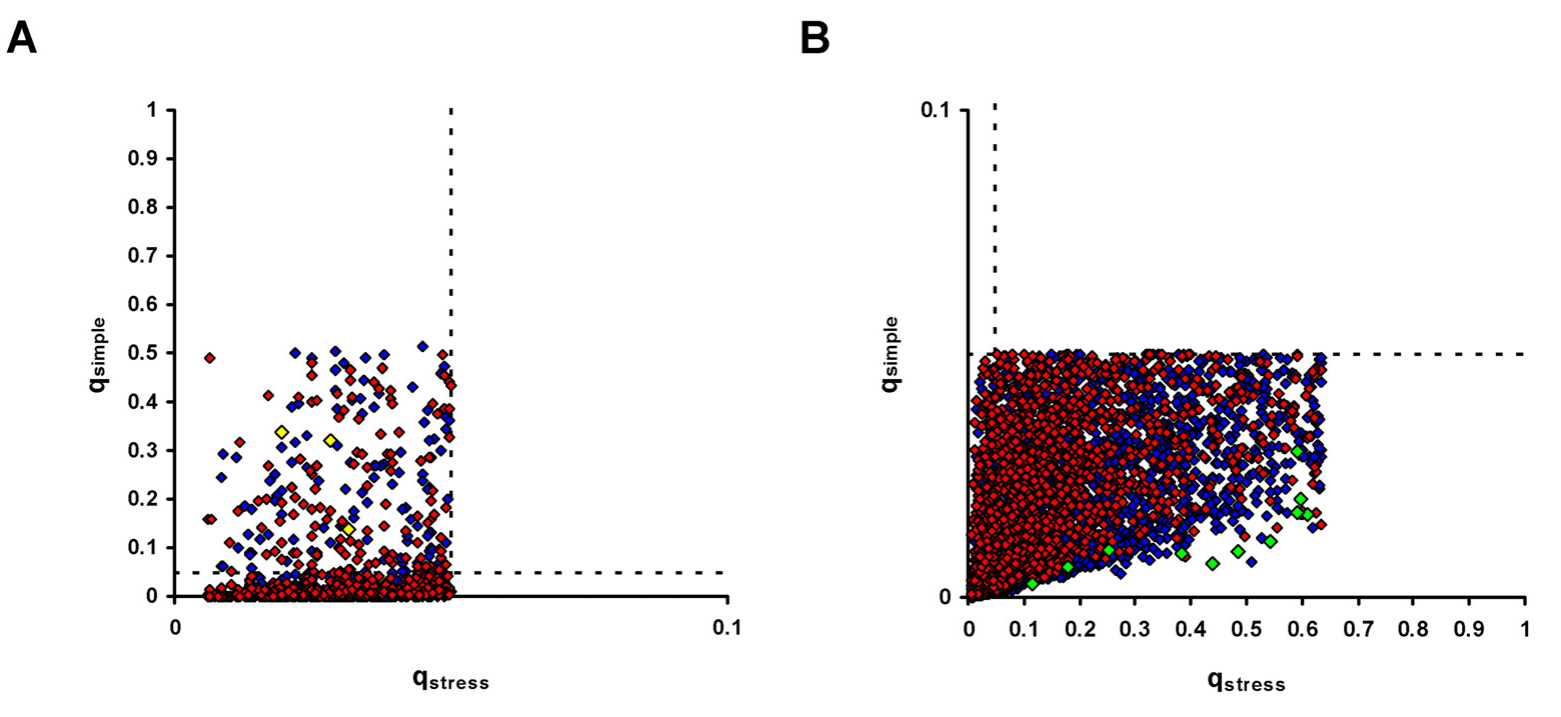
**Comparison of analysis with and without consideration of the stress effect**. For a given feature the q-value for testing differential expression between B and M cells under the stress model (q_stress_) was plotted against the q-value obtained under the simple model (q_simple_). **A** 1,280 unique features selected as q_stress _< 0.05. Dashed lines cut axes at q_stress _= 0.05 and q_simple _= 0.05. **B** 4,384 unique features selected as q_simple _< 0.05. Features predicted to be M enriched by stress or simple models, in **A. **or **B. **respectively, shown in red. Features predicted to be B enriched by stress or simple models, in **A. **or **B. **respectively, shown in blue. Four features annotated as PSII components (MZ00023434, MZ00044083, MZ00040590, MZ00041794), not identified as M enriched by the simple model, are shown in yellow in **A. **Eleven features annotated as heat shock proteins (MZ00000354, MZ00034301, MZ00035916, MZ00035984, MZ00036574, MZ00038036, MZ00039146, MZ00040123, MZ00040558, MZ00040980 and MZ00042904), predicted to be strongly M enriched by the simple model, are shown as green in **B**.

Lists of differentially accumulating features identified by the stress and simple models were compared. Under FDR control at the 5% level with q-values ≤ 0.05, the stress model identified 1,280 differentially accumulating unique features. At the same level of control, the simple model identified 4,384 unique features. 1,043 features were common to the gene lists obtained from the two models. Therefore, the simple model identified the majority of features identified by the stress model (approximately 80%). This is shown graphically in Figure 3A. For the features identified by the stress model, the q value obtained from the stress model (q_stress_) is plotted against the q value obtained from the simple model (q_simple_). Features are coloured red and blue for predicted M and B enrichment, respectively. The threshold values of q = 0.05 are shown by dashed lines. Among the features identified by the stress model, but not the simple model, were three annotated as components of PSII (MZ00023434, MZ00044083, MZ00040590) (shown in yellow in Figure 3A). Previous analyses have shown that PSII components predominantly accumulate in the M cells of the maize leaf [8,9]. The failure of the simple model to identify these PSII transcripts is consistent with reduction in RNA levels associated with M cell preparation. Under the stress model, accumulation of all three of these RNAs was predicted to be reduced by stress.

Although the simple model identified the majority of features identified by the stress model, it also identified many more features in total than the stress model (Figure 3B). Preliminary microarray analysis of B and M cell types using a simple paired comparison had suggested that one confounding effect of preparation stress might be the mistaken identification of stress induced transcripts as M enriched (Sawers *et al*., unpublished observations). Among the features predicted to be strongly M enriched only under the simple model were a number annotated as chaperones or heat shock proteins (HSPs) (MZ00000354, MZ00034301, MZ00035916, MZ00035984, MZ00036574, MZ00038036, MZ00039146, MZ00040123, MZ00040558, MZ00040980 and MZ00042904), a class of transcripts known to be induced by stresses [38,39]. These HSPs are shown in green in Figure 3B. Additional stress-related annotations included a glutathione peroxidase (MZ00041338, MZ00041463) and a wounding-associated chymotrypsin inhibitor (MZ00037253, MZ00041005). These HSPs and other stress-related features are likely to have been mis-identified as M enriched on the basis of stress-related increases in accumulation. Consistent with this interpretation, the stress model predicted that accumulation of all of these transcripts increased following stress.

Empirical comparison of simple and stress models demonstrated the requirement for the control of the stress effect and the applicability of our stress model to this problem and is, therefore, the model we have chosen in the analysis of differential gene expression between B and M cells. Below, we describe the use of the stress model to identify potentially differentially accumulating transcripts and briefly discuss the resulting gene list.

### Statistical identification of B and M enriched transcripts using the stress model

The estimate (*V*_*1f *_- *V*_*2f*_) was calculated for all 15,988 features passing data filtering. The mean estimate of (*V*_*1f *_- *V*_*2f*_) was 0.02 suggesting that the estimates were distributed around a value close to 0, i.e. equivalent expression in B and M cell types. Analysis of the distribution of resulting p-values allowed estimation of the proportion of non-differentially expressed genes (π_0_). Using the method of Storey et al., [37], π_0 _was calculated to be 0.823, corresponding to 2,830 differentially expressed features from the 15,988 in the analysis. Under FDR control at the 5% level, the model identified 1,280 candidate features.

### Annotation of features identified as differentially expressed under FDR control

The majority of features present in the Maize Array Project are designed to EST contigs present in the TIGR maize sequence database [40]. An annotation of the features, based on annotation of the TIGR sequences at the time of design, is available at the Maize Array Project website [41]. It is important to note that a complete maize genome sequence is not yet available and that current gene models may well change as additional data become available. Annotation of oligonucleotide probes is further complicated by difficulty in predicting cross-hybridization between related genes [42,43]. This is especially relevant to a highly polymorphic species such as maize that possesses a highly duplicated genome [44]. Given these caveats, approximately 50% of the identified features were fully or partially annotated in the Maize Array Project database. Of the remainder, approximately 15% were annotated as encoding genes related to hypothetical or unknown *Arabidopsis *or rice proteins, 10% were annotated by similarity to *Arabidopsis *or rice genomic regions and 25% were not annotated. To further investigate the number of unique genes represented by the 1,280 features, a BLAST search was used to identify maize sequences in the NCBI non-redundant database homologous to the oligonucleotides. For each feature, the best-matched sequence was recorded. Under the search criteria used 1,173 matches were recovered. Of these, 899 unique sequences were represented. In addition, TIGR rice gene model matches were obtained for 792 of the features from the Maize Array Project database. These 792 rice matches represent 730 independent gene models. This estimate is in line with the original design criteria of the microarray used [33]. Annotations are included in Additional file 2.

### Validation of candidate gene list using prior knowledge

**Figure 4:**
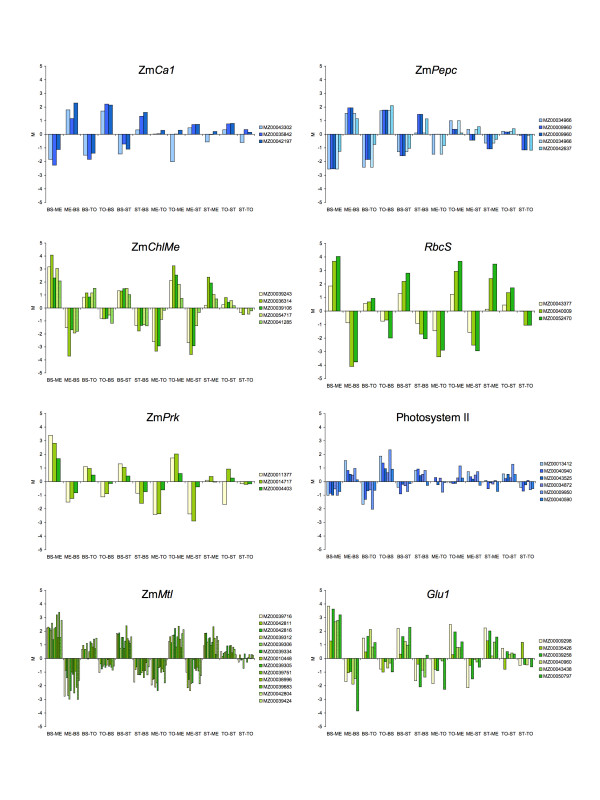
**Accumulation of transcripts encoded by representative C_4 _marker genes**. The ratios of signal intensity (*M *values) for each feature across the twelve hybridizations. Hybridizations are listed as Cy5 sample – Cy3 sample. Positive *M *values correspond to higher signal in the Cy5 channel. Features in green are predicted to be B enriched whereas features in blue are predicted to be M enriched. Features are listed according to MZ identifiers [87]. Zm*Ca1 *(carbonic anhydrase), Zm*Pepc *(phosphoenolpyruvate carboxylase), Zm*ChlMe *(malic enzyme), *RbcS *(Rubisco SSU), Zm*Prk *(phosphoribulokinase), Zm*Mtl *(metallothionein), *Glu1 *(β-glucosidase). The chart 'Photosystem II' shows features corresponding to a number of transcripts: MZ00013412, *psbH*; MZ00040940, *psbH*; MZ00043525, *psbS*; MZ00034872, *psbT*; MZ00009950, *psbW*; MZ00040590, *oee3*.

Differential gene expression in B and M cells has been the subject of extensive prior investigation [18]. The wealth of previous studies provides a collection of well-characterized marker transcripts that can be used in the preliminary validation of the candidate gene list. In total, we identified approximately 80 features as markers considered to provide biological validation of the data set and analysis (Additional file 2). Data corresponding to a number of these are shown graphically in Figure 4. Markers were selected on the basis of previous studies and the presence of multiple features representing a gene. For each feature, the ratios of signal intensity (*M *values) obtained from the 12 hybridizations are shown. While variation is evident both between and among features, the overall consistency of behaviour is evident. In all examples shown, strong B or M cell enrichment is predicted in accordance with previous observations.

The enzymes of the C_4 _carbon shuttle are abundant and cell-type specific (Figure 1B). Previous studies have characterised accumulation of their transcripts by RNA gel-blot analysis [45], *in situ *hybridization [46], real-time PCR [27] and differential screening [26]. Features corresponding to genes encoding the carbon shuttle enzymes, phosphoenolpyruvate carboxylase (PEPC), malate dehydrogenase (MDH), NADP-dependent malic enzyme (NADP-ME) and pyruvate orthophosphate dikinase (PPDK) activities are shown in Figure 4. Although considered markers of C_4 _cell identity, the carbon shuttle enzymes are encoded by members of small gene families that contain both C_3 _and C_4 _isoforms. For example, C_4_-specific malic enzyme is hypothesized to have arisen following the acquisition of a plastid transit peptide sequence by a gene encoding a cytosolic isoform, followed by duplication and divergence of C_3 _and C_4 _forms [47]. Consequently, maize contains at least three NADP-ME loci [47]. The gene Zm*ChlMe1 *encodes a leaf-specific, plastid-targeted isoform required for decarboxylation of malate in B cells [47,48] (Figure 1B, reaction 3) while two, nearly identical, *Me2 *genes (Zm*ChlMe2a *and Zm*ChlMe2b*) have been identified that encode cytosolic isoforms [47]. At the nucleotide level, Zm*ChlMe2a *and Zm*ChlMe2b *are 99% identical to each other and 87% identical to Zm*ChlMe1 *[47]. A further NADP-ME activity has been characterized from roots and found to be 99% and 98% identical to Zm*ChlMe2a *and Zm*ChlMe2b*, respectively [49]. Gene duplications of this type could pose a serious difficulty in oligonucleotide analysis if features do not discriminate between paralogous gene copies. In the case of the carbon shuttle enzymes, the C_4 _isoforms are typically more abundant than C_3 _isoforms [50] and the accumulation patterns we observed suggest that the signal obtained in our experiment corresponds to C_4 _cell-specific transcripts.

A somewhat different situation is illustrated by the multi-subunit Rubisco holoenzyme. Here, an enzyme that is abundant in all photosynthetic cell types in the C_3 _plant is restricted to the B in maize, although the biochemical role of the protein remains unchanged. Rubisco consists of a number of large (LSU) and small (SSU) subunits. LSU is encoded by the chloroplast gene *rbcL *[51], while SSU is encoded by a family of nuclear *RbcS *genes [52-55]. In maize, both LSU and SSU are restricted to the B cells of mature leaf tissue by regulation of transcript accumulation [54-57]. In this experiment, three features were identified corresponding to *RbcS *and each showed the expected B enrichment. Two additional Calvin cycle enzymes, carbonic anhydrase (CA) and phosphoribulokinase (PRK) were also represented by multiple features on the array and displayed the predicted B-enriched patterns of expression (Figure 4).

Previous gene profiling of leaf cell-types in maize [26,58] and sorghum [28] have identified a number of metallothionein (MT) genes that are expressed preferentially in B cells. MTs are a family of small, metal-ion binding proteins that are found in many taxa and are hypothesized to play important roles in metal tolerance and homeostasis [59]. Consistent with these studies, we identified a number of B-enriched features corresponding to MT-like proteins (Figure 3). There are three annotated MT genes in maize, designated Zm*Mtl1 *[60], Zm*Mtl2 *[61] and Zm*Mtl3 *[62]. Although the features we identified showed homology to these genes, the majority were most similar to a non-characterized EST sequence (Gen Bank: CF023010) that we tentatively annotate as Zm*Mtl4*. The role of MT proteins in the B is not immediately apparent. However, the analysis of Nakazono and colleagues [58] suggests an involvement in the functioning of the vasculature.

Proteomic analysis of maize B and M chloroplasts has identified β-glucosidase as among the most strongly B-enriched proteins [63]. Immunocytochemical studies have also demonstrated B enrichment of the major plastidic isoform of β-glucosidase in maize leaves [64,65]. We found six B-enriched features corresponding to β-glucosidase in our candidate gene list. β-glucosidase is proposed to function in plant defence by conversion of hydroxamic acid glucosides to toxic benzoxazolinones [66]. In addition, β-glucosidase activity has been implicated in cytokinin signalling [67]. Although providing a further marker for B identity, the significance of the localization of the enzyme in maize leaves remains unresolved.

In summary, approximately 8% (1,280 of 15,988) of the features analysed were identified as accumulating differentially between B and M when FDR is controlled at 5%. Approximately 50% of these features were fully or partially annotated by the maize array project database. Searching of maize sequences databases suggested that that these features represent at least 899 unique genes. An estimate of the overall predicted proportion of differentially accumulating features (1-π_0_) suggests that as many as 18% of features may be differentially expressed. Approximately 80 features identified in our gene list correspond to previously characterized marker genes and provide convincing evidence of the validity of our data set and analysis. Below we consider the significance of differential expression in the establishment and potential engineering of the C_4 _syndrome.

### General comments on the differentiation of B and M cell types

It is estimated that the C_4 _syndrome has been derived at least 45 times in 19 families of angiosperms [5] and that a small number of regulatory changes are sufficient to establish a functional C_4 _type [68]. By contrast, our observations suggest that differentiation of B and M cell-types in maize involves the cell-specific regulation of many thousands of genes. A number of these differences likely pre-date the acquisition C_4 _photosynthesis. Ancestrally, the M would have been the major site of photosynthesis while the B would have had, and presumably retains, functions associated with proximity to the vascular tissue [69]. Indeed, C_3 _isoforms of certain C_4_-related enzymes show specialised patterns of accumulation in the leaves of C_3 _plants [70]. Additionally, a number of C_4 _cell-specific promoters have been shown to be functional when introduced into C_3 _species [71-76]. These observations suggest that spatial information distinguishing B and M also pre-dates the shift to C_4_.

**Figure 5:**
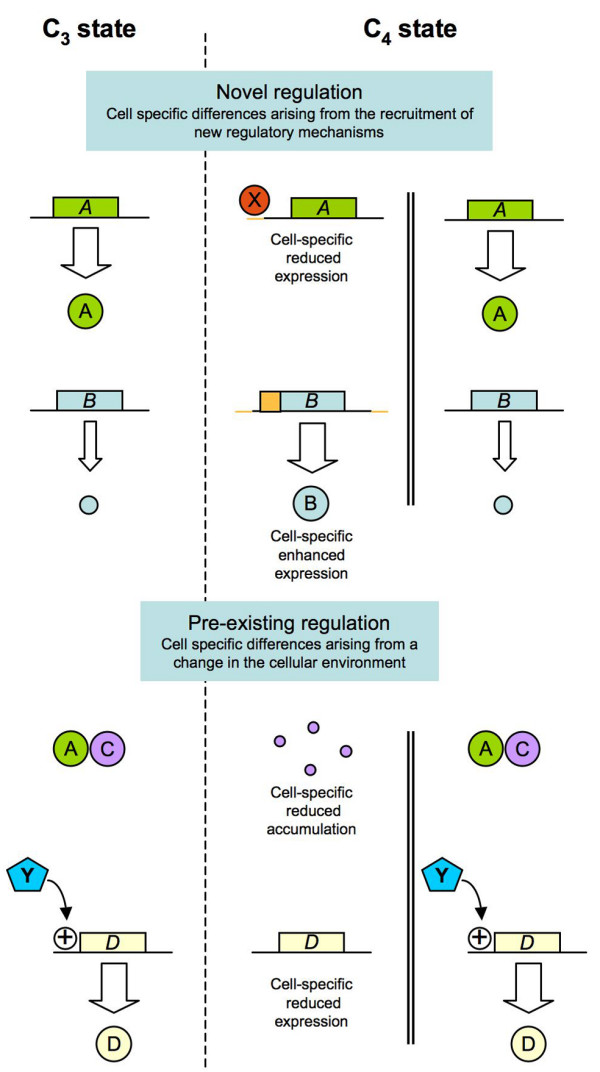
**Mechanisms of differential regulation**. The C_4_-specific regulation of four hypothetical genes and their protein products is illustrated. During the establishment of the C_4 _syndrome, novel regulatory mechanisms that were absent in the C_3 _state control the accumulation of proteins A and B. In the case of A, a protein that accumulates in all photosynthetic cell types in the C_3 _state is restricted to a specific cell type, by a *trans *acting factor X, without any change to biochemical function. Gene B, expressed at low levels, accumulates in a specific cell type. Proteins C and D also accumulate differentially in the C_4 _state, but, in these instances, differential accumulation is the result of a novel cellular environment. In the case of C, protein accumulation is regulated post-translationally at the level of assembly or stability through interaction with A. In the C_4 _state and the absence of A, product C fails to accumulate. Such a scenario does not require differential accumulation of C transcripts, but could affect transcription of other genes. Transcription of gene D is linked, either directly of indirectly, to the presence of a metabolic signal Y. The accumulation of Y is governed by the activities of proteins A and B. In the C_4 _state, changes in the accumulation of A and B affect the levels of Y and subsequently alter the accumulation of D. Boxes represent genes and spheres represent gene products.

Although it is likely there were ancestral differences between B and M, our data and previous studies suggest that the establishment of the C_4 _syndrome resulted in many additional changes to the accumulation of transcripts within these cell types. We distinguish two modes of regulation that might establish these differences (shown graphically in Figure 5). First, modification of *cis*-acting elements and the recruitment of transcription factors may directly change patterns of gene expression (genes A and B in Figure 5). Studies of C_4 _gene regulation in maize have successfully identified such elements associated with a number of genes [16,17,19,77,47]. Although it has been assumed that regulatory changes of this type drive the establishment of the C_4 _state, it appears unlikely that novel regulatory elements could be recruited on the scale required to explain the number of differentially expressed genes we have observed. As a second mechanism, we suggest that pre-existing regulatory mechanisms established in the C_3 _state respond in a cell-specific manner to the creation of novel environments in the C_4 _leaf (genes C and D in Figure 5). The B and M cells of a C_4 _leaf differ in their complement of protein complexes, concentration of sugars, the redox poise of the photosynthetic electron transport chain and the availability of reducing equivalents [78]. More broadly, we suggest that a small number of changes in gene expression, when superimposed on ancestral cellular differences, would further induce secondary changes in transcript accumulation and thereby generate the complex pattern we have observed. It should be noted that such a model does not suggest the presence of regulatory 'master-switches' but rather the re-balancing of a complex system in response to key alterations. We have recently initiated transcriptional profiling of maize mutants with defects in phytochrome signalling [79], Calvin cycle function [22] and tetrapyrrole biosynthesis [80,81] to assess the effect of disrupting cellular conditions on cell-specific patterns of gene expression.

### Engineering C_4 _photosynthesis

The ability to express C_4_-associated transcripts in C_3 _plants has stimulated interest in the molecular engineering of a C_4 _state in C_3 _plants to achieve high photosynthetic performance and water and nitrogen use efficiencies [82,83]. Unfortunately, the physiological effects of over-expressing individual carbon shuttle proteins in C_3 _plants have been limited and difficult to interpret [82]. The next steps in C_4 _engineering will require that multiple enzymes be expressed together. To achieve this, knowledge of the mechanisms that coordinate the distribution of activities in the C_4 _leaf will be essential. In addition, an appreciation of the extent of *de novo *regulation required for the establishment of the C_4 _state will aid the selection of targets for manipulation. Many of the most promising attempts to transfer individual C_4 _activities to a C_3 _plant have been achieved by transfer of maize genes to rice [71-74]. Similarly, the applicability of information regarding genome wide regulatory events will likely be greatest between closely related species. In this regard, comparative genomic and proteomic studies of maize and rice offer perhaps the greatest potential for understanding the establishment of the C_4 _syndrome.

## Conclusion

Differential gene expression was examined in separated B and M cell types from maize leaf blade tissue. To control for stress effects generated during the isolation process we developed a model that includes a stress term and compare the results of the analysis with a model lacking the stress term. These results suggest that gene expression changes are induced during the M cell isolation process and that this confounding effect can be reduced using the stress model. Our analysis indicates that 8% of features detected on the maize long-oligonucleotide microarray produced by the Maize Array Project consortium and up to 18% of genes expressed in the leaf transcriptome are differentially expressed between B and M cell types.

## Methods

### Plant material and growth conditions

Wild-type W22 inbred maize seedlings were grown in a growth chamber at 28°C, 500 μmol m^-2 ^s^-1 ^light, 16 h days, 8 h nights. Light was provided by a combination of 400-W metal halide and 100-W halogen lamps. Ten-day old seedlings were harvested 2 h after the start of the light period for bundle sheath and mesophyll preparation.

### Preparation of bundle sheath strands and mesophyll protoplasts

Bundle sheath strands and mesophyll protoplasts were prepared as previously described [22,84]. Approximately 5 g of tissue were harvested from the second and third leaves of 10-day-old maize seedlings for each mesophyll preparation. Approximately 4 g of tissue were harvested from the second and third leaves for each bundle sheath preparation.

### Preparation of RNA

RNA was prepared as previously described [85]. DNAse treatment was performed using amplification grade DNAse I (Invitrogen, Carlsbad, CA) in the presence of RNase OUT RNase Inhibitor (Invitrogen). Following treatment, RNA was extracted first with phenol:chloroform:IAA (24:1:1) and then with chloroform:IAA (24:1). Following extraction, RNA was ethanol precipitated, washed twice in 70% ethanol and re-suspended in DEPC-treated dH_2_O.

### Microarray detection

Microarray detection was performed using the Genisphere 3DNA 900 MPX two-stage labelling kit (Genisphere, Hatfield, PA). 8 μg of DNAse-treated total RNA were used per labelling reaction and resulting cDNA products split between a two-slide set covering a total footprint area of ~3000 mm^2^. cDNA synthesis was primed using oligo dT and random primers according to the manufacturer's protocol. Maize oligonucleotide microarrays were obtained from the Maize Array Project (University of Arizona) as described [32]. Arrays were imaged using the Scan Array 5000 system (Perkin Elmer, Wellesley, MA). Intermediate laser gain (60–70%) was used to detect a majority of features while minimizing the problem of signal saturation from highly expressed genes.

### Preliminary data processing and background correction

Preliminary segmentation and data extraction were performed using Imagene software (Biodiscovery, El Segundo, CA). For each feature, median signal (SMD) and background (BMD) values were extracted. To correct for background noise, the difference between the log values of SMD and BMD was calculated (Corrected intensity = log_2_(SMD) - log_2_(BMD)) [86]. Corrected data sets were examined graphically by plotting the difference between Cy5 and Cy3 (*M *= corrected intensity Cy5 – corrected intensity Cy3) against the average intensity of Cy5 and Cy3 signals [*A *= (corrected intensity Cy5 + corrected intensity Cy3)/2] for each slide.

### LOWESS normalization and data filtering

A LOWESS procedure [35] was used to normalize signal intensity between channels for every slide. The difference (*M*) and average intensity (*A*) values were calculated as described above. A LOWESS regression was applied to *M *against *A *and the resulting trend-line was used to centralise *M *values around 0 and to correct for any dependence of *M *on *A*. Unreliable or uninformative data were discarded to reduce the dimensionality of the analysis. Data were discarded if intensity measurements were considered either too low (i.e. detection failed) or too high (i.e. signal saturation). Filtering criteria were applied to data obtained from T_T _hybridizations to retain features showing extreme expression profiles only under certain conditions. The background corrected intensities were averaged across the six T_T _hybridization data sets. A feature was discarded if this average was less than 1 (i.e. geometric average SMD < 2xBMD). Additionally, a feature was discarded as saturating if 3 or more out of the 6 T_T _signal intensities read at the maximum. Following data filtering, 47,591 features were discarded from an original 64,896 because of low expression. Of the remainder, a further 178 features were removed because of saturation. Excluding the control spots, we have a total of 15,988 unique features (25% of the original set) for subsequent analysis. The normalized *M *values for these 15,988 features are provided in Additional file 1.

## Authors' contributions

RS designed the study and analytical models, performed experimental procedures and data extraction and drafted the manuscript. PL and GH performed data analysis, including development and implementation of analytical models. KA assisted in the preparation of isolated cell-types and the extraction of RNA. TB contributed to the experimental design and drafting of the manuscript. All authors have read and approved the final manuscript.

## Supplementary Material

Additional file 1 **Normalised M values for features passing data-filtering**. Feature list spreadsheet. *M *values are provided for each of 12 slides. See Methods for details. Click here for file

Additional file 2 **Estimates of (*v1-v2*) under stress model and annotation for features passing data-filtering**. Feature list spreadsheet. (*v1-v2*) estimate of the log normalised difference between B (*v1*) and M (*v2*) expression derived from the stress model described in the text. FOLD is the degree of enrichment as calculated by back-transformation of the (*v1-v2*) estimate. Click here for file

Additional file 3 **Estimates of (*v1-v2*) under simple model and annotation for features passing data-filtering**. Feature list spreadsheet. (*v1-v2*) estimate of the log normalised difference between B (*v1*) and M (*v2*) expression derived from the simple model described in the text without stress term. FOLD is the degree of enrichment as calculated by back-transformation of the (*v1-v2*) estimate under simple model or stress model as labeled. Click here for file

## References

[B1] Sage RF, Monson RK (1999). C4 plant biology.

[B2] Bowes G, Ogren WL, Hageman RH (1971). Phosphoglycolate production catalyzed by ribulose diphosphate carboxylase. Biochem Biophys Res Commun.

[B3] Orgren WL (1984). Photorespiration: pathways, regulation and modification.. Annu Rev Plant Physiol.

[B4] Moore P (1982). Evolution of photosynthetic pathways in flowering plants. Nature.

[B5] Sage RF (2004). The evolution of C4 photosynthesis. New Phytol.

[B6] Brown NJ, Parsley K, Hibberd JM (2005). The future of C4 research--maize, Flaveria or Cleome?. Trends Plant Sci.

[B7] Meierhoff K, Westhoff P (1993). Differential biogenesis of photosystem II in mesophyll and bundle sheath cells of monocotyledonous NADP-malic enzyme type C4 plants: The non-stoichiometric adundance of the subunits of photosystem II in the bundle sheath chloroplasts and the translational activity of the plastome encoded genes. Planta.

[B8] Schuster G, Ohad I, Martineau B, Taylor WC (1985). Differentiation and development of bundle sheath and mesophyll thylakoids in maize. Thylakoid polypeptide composition, phosphorylation, and organization of photosystem II. J Biol Chem.

[B9] Sheen J, Bogorad L (1988). Differential expression in bundle sheath and mesophyll cells of maize of genes for photosystem II components encoded by the plastid genome. Plant Physiol.

[B10] Hatch MD (1992). C4 photosynthesis: an unlikely process full of surprises. Plant Cell Physiol.

[B11] Leegood RC (1997). The regulation of C4 photosynthesis. Adv Bot Res.

[B12] Doulis AG, Debian N, Kingston-Smith AH, Foyer CH (1997). Differential localization of antioxidants in maize leaves. Plant Physiol.

[B13] Rathnam CK, Edwards GE (1976). Distribution of nitrate-assimilating enzymes between mesophyll protoplasts and bundle sheath cells in leaves of three groups of C(4) plants. Plant Physiol.

[B14] Langdale JA, Taylor WC, Nelson T (1991). Cell-specific accumulation of maize phosphoenolpyruvate carboxylase is correlated with demethylation at a specific site greater than 3 kb upstream of the gene. Mol Gen Genet.

[B15] Schaffner AR, Sheen J (1991). Maize rbcS promoter activity depends on sequence elements not found in dicot rbcS promoters. Plant Cell.

[B16] Sheen J (1991). Molecular mechanisms underlying the differential expression of maize pyruvate, orthophosphate dikinase genes. Plant Cell.

[B17] Schaffner AR, Sheen J (1992). Maize C4 photosynthesis involves differential regulation of phosphoenolpyruvate carboxylase genes. Plant J.

[B18] Sheen J (1999). C4 Gene Expression. Annu Rev Plant Physiol Plant Mol Biol.

[B19] Xu T, Purcell M, Zucchi P, Helentjaris T, Bogorad L (2001). TRM1, a YY1-like suppressor of rbcS-m3 expression in maize mesophyll cells. Proc Natl Acad Sci U S A.

[B20] Hall LN, Rossini L, Cribb L, Langdale JA (1998). GOLDEN 2: a novel transcriptional regulator of cellular differentiation in the maize leaf. Plant Cell.

[B21] Hall LN, Roth R, Brutnell TP, Langdale JA (1998). Cellular differentiation in the maize leaf is disrupted by bundle sheath defective mutations. Symp Soc Exp Biol.

[B22] Brutnell TP, Sawers RJ, Mant A, Langdale JA (1999). BUNDLE SHEATH DEFECTIVE2, a novel protein required for post-translational regulation of the rbcL gene of maize. Plant Cell.

[B23] Sheen J (1995). Methods for mesophyll and bundle sheath cell separation. Methods Cell Biol.

[B24] Nelson T, Freeling M, Walbot V (1994). Preparation of DNA and RNA from leaves: expanded blades and separated bundel sheath and mesophyll cells. The maize handbook.

[B25] Ishii S (1988). Factors influencing protoplast viability of suspension-cultured rice cells during isolation process. Plant Physiol.

[B26] Furumoto T, Hata S, Izui K (2000). Isolation and characterization of cDNAs for differentially accumulated transcripts between mesophyll cells and bundle sheath strands of maize leaves. Plant Cell Physiol.

[B27] Hahnen S, Joeris T, Kreuzaler F, Peterhansel C (2003). Quantification of photosynthetic gene expression in maize C(3) and C(4) tissues by real-time PCR. Photosynth Res.

[B28] Wyrich R, Dressen U, Brockmann S, Streubel M, Chang C, Qiang D, Paterson AH, Westhoff P (1998). The molecular basis of C4 photosynthesis in sorghum: isolation, characterization and RFLP mapping of mesophyll- and bundle-sheath-specific cDNAs obtained by differential screening. Plant Mol Biol.

[B29] Clarke JD, Zhu T (2006). Microarray analysis of the transcriptome as a stepping stone towards understanding biological systems: practical considerations and perspectives. Plant J.

[B30] Markelz NH, Costich DE, Brutnell TP (2003). Photomorphogenic responses in maize seedling development. Plant Physiol.

[B31] Kerr MK, Churchill GA (2001). Experimental design for gene expression microarrays. Biostatistics.

[B32] Maize Oligonucleotide Array Project. http://www.maizearray.org/index.shtml.

[B33] Gardiner JM, Buell CR, Elumalai R, Galbraith DW, Henderson DA, Iniguez AL, Kaeppler SM, Kim JJ, Liu J, Smith A, Zheng L, Chandler VL (2005). Design, production, and utilization of long oligonucleotide microarrays for expression analysis in maize.. Maydica.

[B34] Maize Oligonucleotide Array Studies. http://www.maizearray.org/tigr-scripts/maizearray/study/maize_study_hybs.pl?study=4&user=&pass=&sort=id&order=asc.

[B35] Dudoit S, Yang YH, Callow MJ, Speed TP (2002). Statistical methods for identifying differentially expressed genes in replicated cDNA microarray experiments. Statistica Sinica.

[B36] Cui X, Hwang JT, Qiu J, Blades NJ, Churchill GA (2005). Improved statistical tests for differential gene expression by shrinking variance components estimates. Biostatistics.

[B37] Storey JD, Taylor JE, Siegmund D (2004). Strong control, conservative point estimation and simultaneous conservative consistency of false discovery rates: A unified approach. Journal of the Royal Society, Series B.

[B38] Oono Y, Seki M, Nanjo T, Narusaka M, Fujita M, Satoh R, Satou M, Sakurai T, Ishida J, Akiyama K, Iida K, Maruyama K, Satoh S, Yamaguchi-Shinozaki K, Shinozaki K (2003). Monitoring expression profiles of Arabidopsis gene expression during rehydration process after dehydration using ca 7000 cDNA microarray. Plant J.

[B39] Seki M, Narusaka M, Ishida J, Nanjo T, Fujita M, Oono Y, Kamiya A, Nakajima M, Enju A, Sakurai T, Satou M, Akiyama K, Taji T, Yamaguchi-Shinozaki K, Carninci P, Kawai J, Hayashizaki Y, Shinozaki K (2002). Monitoring the expression profiles of 7000 Arabidopsis genes under drought, cold and high-salinity stresses using a full-length cDNA microarray. Plant J.

[B40] The TIGR Maize Database. http://maize.tigr.org/.

[B41] Maize Oligonucleotide Array Search. http://www.maizearray.org/maize_search_basic.shtml.

[B42] Lee I, Dombkowski AA, Athey BD (2004). Guidelines for incorporating non-perfectly matched oligonucleotides into target-specific hybridization probes for a DNA microarray. Nucleic Acids Res.

[B43] SantaLucia J (1998). A unified view of polymer, dumbbell, and oligonucleotide DNA nearest-neighbor thermodynamics. Proc Natl Acad Sci U S A.

[B44] Ma J, Morrow DJ, Fernandes J, Walbot V (2006). Comparative profiling of the sense and antisense transcriptome of maize lines. Genome Biol.

[B45] Sheen JY, Bogorad L (1987). Differential expression of C4 pathway genes in mesophyll and bundle sheath cells of greening maize leaves. J Biol Chem.

[B46] Langdale JA, Rothermel BA, Nelson T (1988). Cellular pattern of photosynthetic gene expression in developing maize leaves. Genes Dev.

[B47] Tausta SL, Coyle HM, Rothermel B, Stiefel V, Nelson T (2002). Maize C4 and non-C4 NADP-dependent malic enzymes are encoded by distinct genes derived from a plastid-localized ancestor. Plant Mol Biol.

[B48] Rothermel BA, Nelson T (1989). Primary structure of the maize NADP-dependent malic enzyme. J Biol Chem.

[B49] Saigo M, Bologna FP, Maurino VG, Detarsio E, Andreo CS, Drincovich MF (2004). Maize recombinant non-C4 NADP-malic enzyme: a novel dimeric malic enzyme with high specific activity. Plant Mol Biol.

[B50] Svensson P, Blasing OE, Westhoff P (1997). Evolution of the enzymatic characteristics of C4 phosphoenolpyruvate carboxylase--a comparison of the orthologous PPCA phosphoenolpyruvate carboxylases of Flaveria trinervia (C4) and Flaveria pringlei (C3). Eur J Biochem.

[B51] Coen DM, Bedbrook JR, Bogorad L, Rich A (1977). Maize chloroplast DNA fragment encoding the large subunit of ribulosebisphosphate carboxylase. PNAS.

[B52] Matsuoka M, Kano-Murakami Y, Tanaka Y, Ozeki Y, Yamamoto N (1987). Nucleotide sequence of cDNA encoding the small subunit of ribulose-1,5-bisphosphate carboxylase from maize. J Biochem (Tokyo).

[B53] Lebrun M, Waksman G, Feyssinet G (1987). Nucleotide sequence of a gene encoding corn ribulose-1,5-bisphosphate carboxylase/oxygenase small subunit (rbcs).. Nucleic Acids Res.

[B54] Sheen J, Bogorad L (1986). Expression of the ribulose-1,5-bisphosphate carboxylase large subunit gene and three small subunit genes in two cell types of maize leaves. Embo J.

[B55] Ewing RM, Jenkins GI, Langdale JA (1998). Transcripts of maize RbcS genes accumulate differentially in C3 and C4 tissues. Plant Mol Biol.

[B56] Link G, Coen DM, Bogorad L (1978). Differential expression of the gene for the large subunit of ribulose bisphosphate carboxylase in maize leaf cell types. Cell.

[B57] Huber SC, Hall TN, Edwards GE (1976). Differential Localization of Fraction I Protein between Chloroplast Types. Plant Physiol.

[B58] Nakazono M, Qiu F, Borsuk LA, Schnable PS (2003). Laser-capture microdissection, a tool for the global analysis of gene expression in specific plant cell types: identification of genes expressed differentially in epidermal cells or vascular tissues of maize. Plant Cell.

[B59] Robinson NJ, Tommey AM, Kuske C, Jackson PJ (1993). Plant metallothioneins. Biochem J.

[B60] de Framond AJ (1991). A metallothionein-like gene from maize (Zea mays). Cloning and characterization. FEBS Lett.

[B61] White CN, Rivin CJ (1995). Characterization and expression of a cDNA encoding a seed-specific metallothionein in maize. Plant Physiol.

[B62] Charbonnel-Campaa L, Lauga B, Combes D (2000). Isolation of a type 2 metallothionein-like gene preferentially expressed in the tapetum in Zea mays. Gene.

[B63] Majeran W, Cai Y, Sun Q, van Wijk KJ (2005). Functional differentiation of bundle sheath and mesophyll maize chloroplasts determined by comparative proteomics. Plant Cell.

[B64] Nikus J, Jonsson LMV (1999). Tissue localization of B-glucosidase in rye, maize and wheat seedlings. Physiol Plant.

[B65] Nikus J, Daniel G, Jonsson LMV (2001). Subcellular loclization of B-glucosidase in rye, maize and wheat seedlings. Physiol Plant.

[B66] Niemeyer HM (1988). Hyroxamic acids (4-hydroxy-1,4-benzoxazin-3-ones) defense chemicals in the Gramineae. Phytochem.

[B67] Brzobohaty B, Moore I, Kristoffersen P, Bako L, Campos N, Schell J, Palme K (1993). Release of active cytokinin by a beta-glucosidase localized to the maize root meristem. Science.

[B68] Ku MS, Kano-Murakami Y, Matsuoka M (1996). Evolution and expression of C4 photosynthesis genes. Plant Physiol.

[B69] Dengler N, Nelson T, Sage RF, Monson RK (1999). Leaf structure and development in C4 plants. C4 Plant Biology.

[B70] Hibberd JM, Quick WP (2002). Characteristics of C4 photosynthesis in stems and petioles of C3 flowering plants. Nature.

[B71] Matsuoka M, Kyozuka J, Shimamoto K, Kano-Murakami Y (1994). The promoters of two carboxylases in a C4 plant (maize) direct cell-specific, light-regulated expression in a C3 plant (rice). Plant J.

[B72] Matsuoka M, Sanada Y (1991). Expression of photosynthetic genes from the C4 plant, maize, in tobacco. Mol Gen Genet.

[B73] Ku MS, Agarie S, Nomura M, Fukayama H, Tsuchida H, Ono K, Hirose S, Toki S, Miyao M, Matsuoka M (1999). High-level expression of maize phosphoenolpyruvate carboxylase in transgenic rice plants. Nat Biotechnol.

[B74] Tsuchida H, Tamai T, Fukayama H, Agarie S, Nomura M, Onodera H, Ono K, Nishizawa Y, Lee BH, Hirose S, Toki S, Ku MS, Matsuoka M, Miyao M (2001). High level expression of C4-specific NADP-malic enzyme in leaves and impairment of photoautotrophic growth in a C3 plant, rice. Plant Cell Physiol.

[B75] Svensson P, Blasing OE, Westhoff P (2003). Evolution of C4 phosphoenolpyruvate carboxylase. Arch Biochem Biophys.

[B76] Gowik U, Burscheidt J, Akyildiz M, Schlue U, Koczor M, Streubel M, Westhoff P (2004). cis-Regulatory elements for mesophyll-specific gene expression in the C4 plant Flaveria trinervia, the promoter of the C4 phosphoenolpyruvate carboxylase gene. Plant Cell.

[B77] Metzler MC, Rothermel BA, Nelson T (1989). Maize NADP-malate dehydrogenase: cDNA cloning, sequence, and mRNA characterization.. Plant Mol Biol.

[B78] Kanai R, Edwards G, Sage RF, Monson RK (1999). The biochemistry of C4 photosynthesis. C4 Plant Biology.

[B79] Sheehan MJ, Kennedy LM, Costich DE, Brutnell TP (2007). Subfunctionalization of PhyB1 and PhyB2 in the control of seedling and mature plant traits in maize.. Plant J.

[B80] Sawers RJ, Linley PJ, Gutierrez-Marcos JF, Delli-Bovi T, Farmer PR, Kohchi T, Terry MJ, Brutnell TP (2004). The Elm1 (ZmHy2) gene of maize encodes a phytochromobilin synthase. Plant Physiol.

[B81] Sawers RJ, Viney J, Farmer PR, Bussey RR, Olsefski G, Anufrikova K, Hunter CN, Brutnell TP (2006). The maize Oil yellow1 (Oy1) gene encodes the I subunit of magnesium chelatase. Plant Mol Biol.

[B82] Matsuoka M, Furbank RT, Fukayama H, Miyao M (2001). Molecular Engineering of C4 Photosynthesis. Annu Rev Plant Physiol Plant Mol Biol.

[B83] Mitchell PL, Sheehy JE (2006). Supercharging rice photosynthesis to increase yield. New Phytol.

[B84] Westhoff P, Offermann-Steinhard K, Hofer M, Eskins K, Oswald A, Streubel M (1991). Differential accumulation of plastidic transcripts encoding photosystem II components in the mesophyll and bundle sheath cells of monocotylednonous NADP-malic enzyme-type C4 plants. Planta.

[B85] Sawers RJ, Linley PJ, Farmer PR, Hanley NP, Costich DE, Terry MJ, Brutnell TP (2002). Elongated mesocotyl1, a phytochrome-deficient mutant of maize. Plant Physiol.

[B86] Zhang D, Zhang M, Wells MT (2006). Multiplicative background correction for spotted microarrays to improve reproducibility. Genet Res.

[B87] Maize Oligonucleotide Array Overview. http://www.maizearray.org/maize_search_overview.shtml.

